# Draft Genome Sequence and Assembly of a *Lysobacter enzymogenes* Strain with Biological Control Activity against Root Knot Nematodes

**DOI:** 10.1128/genomeA.00271-17

**Published:** 2017-05-04

**Authors:** Iker Hernández, Carolina Fernàndez

**Affiliations:** Futureco Bioscience, Olèrdola (Barcelona), Spain

## Abstract

*Lysobacter enzymogenes* strain B25, an isolate from an agricultural field, acts as a biological control agent against root knot nematodes in tomato plants. B25 also controls several fungal diseases and promotes plant growth under abiotic stress. We hereby report on the draft genome sequence and assembly of B25.

## GENOME ANNOUNCEMENT

*Lysobacter enzymogenes* is a bacterium species (class *Gammaproteobacteria*; family *Xanthomonadaceae*) with outstanding extracellular lytic metabolism (1). This species is widely described as a biological control agent (BCA) against fungal plant pathogens (2). Similarly, this species restrains nematode populations *in vitro* (3). In a survey aimed at identifying BCAs against plant parasitic nematodes, we identified *L. enzymogenes* strain B25 from an agricultural field in Gavà (northeast Spain). B25 strongly hinders root knot nematode (*Meloidogyne* spp.) egg hatching and infective juvenile (J2) survival *in vitro* and *in vivo* (4). In addition, B25 promotes plant growth (4) and protects plants from several fungal pathogens, as reported for other *L. enzymogenes* strains (2). We hereby report on the draft genome sequence and assembly of *L. enzymogenes* B25.

The DNA, isolated from a liquid culture initiated from a single B25 colony, was submitted to Genomix4life (Baronissi, Italy) for library preparation (Nextera DNA library prep kit, Illumina, Cambridge, UK) and sequencing (2 × 150 paired-end [PE]; NextSeq 500; Illumina). The raw reads were quality-trimmed using Trim Galore! (5), and subsequently assembled using SPAdes (6). The assembly was constrained to scaffolds longer than 200 nt, with a Blast hit in a *Lysobacter* spp. accession (7), and coverage depth higher than 10. The selected scaffolds were ordered with Mauve Contig Mover (8) using the *L. enzymogenes* C3 draft genome as reference (accession number NZ_CP013140.1). The ordered scaffolds were rescaffolded using SSPACE (9). All tools, except for Blast and Mauve, were implemented through Galaxy servers [Main (10), GVL (11), and VirAmp (12)]. The draft genome was annotated using RAST (13, 14). To overcome the read collapse observed in the *rrn* operon region due to its repetitive nature, the *rrn* copy number of B25 was estimated comparing the coverage depth in the 16S gene with that of a set of single copy genes (15, 16).

The sequencing experiment yielded 4,507,278 raw read pairs. After trimming, 8,140,935 reads remained (3,823,999 PE and 492,937 single-end). The assembly consists of 6,306,554 bases in 261 scaffolds with a 69.9% G+C content, *N*_50_ of 53,391, and *L*_50_ of 38. The average coverage is 206.11 with a standard deviation of 96.99 and a breadth of coverage 0.9439. The annotation predicts a single *rrn* copy, and the coverage depth approach predicts 1.75 copies; the C3 genome shows 2 *rrn* copies so, although most likely this is the case for B25, empirical verification is needed. The annotation predicts 5,186 coding DNA sequences (CDS) in 5,208,396 bases (i.e., 82.6% of the genome). Genome alignments show that B25 shows 5.41% missing and 7.13% extra bases when compared to C3.

### Accession number(s).

This whole-genome shotgun project has been deposited at DDBJ/ENA/GenBank under the accession number MTAY00000000. The version described in this paper is the first version, MTAY01000000.
